# The development of a post-mortem interval estimation for human remains found on land in the Netherlands

**DOI:** 10.1007/s00414-017-1700-9

**Published:** 2017-11-06

**Authors:** H. T. Gelderman, L. Boer, T. Naujocks, A. C. M. IJzermans, W. L. J. M. Duijst

**Affiliations:** 1 Public Health Service IJsselland, Department of Forensic Medicine, Zeven Alleetjes 1, 8011 CV Zwolle, The Netherlands; 2 Public Health Service Groningen, Department of Forensic Medicine, Hanzeplein 120, 9713 GW Groningen, The Netherlands; 3 Public Health Service Hart voor Brabant, Department of Forensic Medicine, Vogelstraat 2, 5212 VL ‘s-Hertogenbosch, The Netherlands; 4 Maastricht University, faculty of law and criminology, Minderbroedersberg 4-6, 6211 LK Maastricht, The Netherlands

**Keywords:** Forensic science, Forensic taphonomy, Post-mortem interval, Decomposition process, Decomposition phenomena, Accumulated degree days

## Abstract

The decomposition process of human remains can be used to estimate the post-mortem interval (PMI), but decomposition varies due to many factors. Temperature is believed to be the most important and can be connected to decomposition by using the accumulated degree days (ADD). The aim of this research was to develop a decomposition scoring method and to develop a formula to estimate the PMI by using the developed decomposition scoring method and ADD.

A decomposition scoring method and a Book of Reference (visual resource) were made. Ninety-one cases were used to develop a method to estimate the PMI. The photographs were scored using the decomposition scoring method. The temperature data was provided by the Royal Netherlands Meteorological Institute. The PMI was estimated using the total decomposition score (TDS) and using the TDS and ADD. The latter required an additional step, namely to calculate the ADD from the finding date back until the predicted day of death.

The developed decomposition scoring method had a high interrater reliability. The TDS significantly estimates the PMI (*R* ^2^ = 0.67 and 0.80 for indoor and outdoor bodies, respectively). When using the ADD, the *R* ^2^ decreased to 0.66 and 0.56.

The developed decomposition scoring method is a practical method to measure decomposition for human remains found on land. The PMI can be estimated using this method, but caution is advised in cases with a long PMI. The ADD does not account for all the heat present in a decomposing remain and is therefore a possible bias.

**Electronic supplementary material:**

The online version of this article (10.1007/s00414-017-1700-9) contains supplementary material, which is available to authorized users.

## Introduction

When human remains are found, the time of death has to be determined. The time between death and finding a body is called the post-mortem interval (PMI). Determining the PMI is important, because having a time frame can help with the identification of the human remains and contribute to investigate the possible causes of death [1]. Several methods have been developed to determine the time of death, for example the Henssge-nomogram and the electrical or mechanical stimulation of skeletal muscles [2–4]. These three methods are only useful in cases with a short PMI, because when a body has attained the ambient temperature, the body cooling stops. The electric and mechanical stimulations of muscles are both usable up to 13 hours post-mortem [2, 3]. Entomology is considered to be one of the most accurate ways to determine the post-mortem interval [5] and is usable in cases with a short PMI as well as a long PMI. In general, it is said that the longer the PMI, the more difficult it is to determine the time of death [6].

Decomposition is a continuous process and is influenced by multiple factors. The influence of these factors on the decomposition is not completely understood yet [7]. Climatic differences between geographical regions, such as a humid or arid, or a hot or cold environment, influence the decomposition [8]. It is known from literature that temperature [5, 9], humidity [10–12], insects [13–16], body weight [7, 14, 17–19] and clothing [20, 21] also have an effect on the decomposition process. It is said that ambient temperature is the most important variable, because it is the primary factor for all biological activity and biochemical reactions [10, 11, 22]. The warmer the ambient temperature, the faster the decomposition is [23]. An environment temperature between 25 and 35 °C is an ideal situation for bacteria to develop [10]. If the aforementioned variables are not included when determining the PMI; the PMI could be longer or shorter than predicted [11].

In multiple studies, the association between decomposition stadia and time was described [24–26]. Galloway et al. [24] divided the decomposition process in five stages. Megyesi et al. [5] modified these stages into a decomposition scoring method and connected the decomposition to temperature by using accumulated degree days (ADD). The ADD is the sum of the average daily ambient temperatures between the date of death and the date of being found. It represents the heat energy units needed for the biological and chemical reactions to decompose a body [14]. Using the decomposition scoring method and ADD, Megyesi et al. [5] have developed a formula which predicted the PMI by 84% (*R* ^2^ value). The scoring method made by Megyesi et al. was validated by Dabbs et al. and Nawrocka et al. who both found a high reliability for the overall total body score, but also made suggestions for improvement [5, 27, 28].

Because a forensic autopsy is seldom performed on human remains in the Netherlands, the developed decomposition method had to be usable without performing an autopsy. Thereby, there was a need to develop a method which could lead to fast decision-making in the field. The first aim of this research was the development of a validated decomposition scoring method which is simple, practical and user friendly. The second aim was to develop a formula to estimate the PMI using the developed decomposition scoring method and ADD.

## Materials and methods

### Decomposition scoring method

A decomposition scoring method, consisting of a decomposition score and a Book of Reference, was made. The Book of Reference is a visual resource containing photographs of the decomposition phenomena. The decomposition scoring method developed by Megyesi et al. [5] was used as a basis. The scoring method separates the human body into three regions (the face and neck, the body and the limbs), because these body parts decompose in a different way [5]. Each body region was assigned six stadia with corresponding scores (see Table 1), with a lowest score of one (no visible changes) and the highest six (complete skeletonization). Every stage contains specific decomposition phenomena but these were not assigned a separate score, because they can occur at the same time or one by one [11, 24]. The total decomposition score (TDS) represents the sum of the facial decomposition score (FDS), the body decomposition score (BDS) and the limbs decomposition score (LDS). The stages of decomposition were derived from an extensive literature study.

Twelve participants (four forensic physicians, four forensic scientists and four medical students) were recruited for the validation test. The decomposition scoring method was explained using the Book of Reference. Forty-five coloured photographs, provided by the Public Health Service (PHS) IJsselland, were equally divided over FDS, BDS and LDS and were shown in a randomised order. The score representing the stage furthest along in the decomposition process had to be assigned to the photographs. Once the validation started, the Book of Reference was not shown again.

The interobserver variability was measured using the Fleiss kappa test via the Online Kappa Calculator [29].

### Selection of cases

**Fig. 1:**
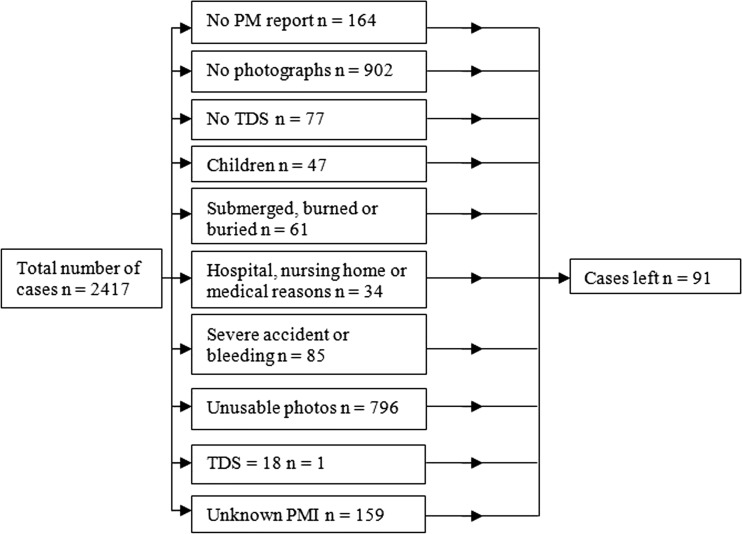
The general selection of cases. TDS=total decomposition score, PM=post-mortem, PMI=post-mortem interval

From the years 2001 until 2016, there were a total of 2417 post-mortem investigations at the PHS IJsselland and PHS Drenthe. To be included, the personal data of the human remains had to be known and a post-mortem report had to be available. Cases had to be closed, which means there was no ongoing investigation. The human remains had to be completed and photographed. Children of 18 years and younger were excluded because of the effects on decomposition of smaller size [6, 7]. There are multiple studies which found faster decay in larger bodies [10, 11, 17, 18] and in contrast, there are also multiple studies which found faster decay in smaller bodies [6, 14, 19]. Children have a different distribution in body surface area compared to adults by having proportionally larger heads and smaller lower extremities [30]. Also, children have a larger body surface area—body content ratio compared to adults. With ageing, the body surface area—body content ratio decreases [31]. Submerged, burned or buried human remains were excluded, because they decompose differently [32–34]. Individuals who passed away in a hospital or nursing home because of medical reasons or after a severe accident were excluded, because often no decomposition phenomena are visible yet. Cases with a TDS of 18 (skeletonization) were excluded, because there is no soft tissue left. A TDS of 18 means the final stage was reached and it is unknown when this stage was reached. After this selection, 250 cases were left and hereof the PMI was known in 91 cases. These 91 cases were used for research (see Fig. 1). The PMI was based on bystanders who were present at the time of death (for example ambulance personnel), last used newspaper or tv-guide, the mail in the mailbox, the last time a person was in contact or was seen or the last time an individual used social media. The PMI was reported in whole days.

### Estimating the post-mortem interval using the decomposition scoring method and ADD

The cases provided by the PHS IJsselland were scored by a forensic physician from the PHS Drenthe and one from the PHS IJsselland. The cases provided by the PHS Drenthe were scored by another forensic physician from the PHS IJsselland. The forensic physicians had no knowledge of the cases they scored and a possible bias was prevented this way. The validated decomposition scoring method was explained to the participants using the Book of Reference before the scores were assigned to the photographs. After the scoring had started, the Book of Reference was not shown again. Photographs were assigned the score representing the decomposition stadia, visible to the eye, which is furthest along in the decomposition process.

Temperature data was needed to determine the ADD. Temperature was not always known when individuals were found indoors. When unknown, an average temperature of 18 °C was filled in. This is considered a normal temperature indoors, with a maximum of 21 °C at day and a minimum of 15 °C at night. For human remains found outside, the temperature data was provided by the Royal Netherlands Meteorological Institute [35]. Weather stations closest to the place of death were used, so that the data was representative for that particular location. Temperatures below zero were not recorded as negative values, but as zero. It is unknown at which temperature the decomposition process stops. Micozzi [36] states that no putrefaction occurs at temperatures below 4 °C while Vass et al. [22] state it is below 0 °C. It was chosen to use 0 °C as a minimum since it is certain that the decomposition process stops while it is freezing.

The statistical analysis was conducted using SPSS, version 23.0 (SPSS Inc.). The normality was tested using the Kolmogorov-Smirnov test. The Spearman correlation coefficient was used to test the correlation between the TDS and PMI. The PMI was estimated via two methods which were compared in this research. The first method consisted of using the TDS to estimate the PMI. The second method consisted of using the TDS to estimate the ADD. The latter required an additional step to estimate the PMI, namely to sum the mean actual daily temperatures from the finding date back until the estimated ADD is reached. For both methods, a linear regression analysis was used. Here, both the PMI and ADD were naturally log transformed to obtain a linear relationship between the TDS and PMI and between the TDS and ADD.

### Ethics and legal

This research was conducted at the PHS IJsselland and PHS Drenthe. A PHS is one of the multiple institutions where forensic physicians work. Forensic physicians perform external post-mortem investigations and do *not* have the legal right to perform an autopsy in the Netherlands. This right is solely for the (forensic) pathologists. The reports are archived together with the available (digital) photographs.

This study was approved by the PHS IJsselland and performed according to the ethical and legal standards in the Netherlands. All data were processed anonymously. For this type of study, formal consent is not required.

## Results

### Decomposition scoring method

Table 1 shows the developed decomposition scoring method. This method was developed after an extensive literature study. Each stage was assigned a score. Each body part had a maximum score of six and the TDS ranges between 3 and 18.

**Table 1:** Developed decomposition scoring method

	Points	Description
FDS	1	1.1 No visible changes
	2	2.1 Livor mortis, rigour mortis and vibices
		2.2 Eyes: cloudy and/or tache noir
		2.3 Discoloration: brownish shades particularly at the edges. Drying of nose, ears and lips
	3	3.1 Grey to green discoloration
		3.2 Bloating of neck and face is present and/or skin blisters, skin slippage and/or marbling
		3.3 Purging of decompositional fluids out of ears, nose and mouth and/or brown to black discoloration
	4	4.1 Caving in of the flesh and tissues of eyes and throat. Skin having a leathery appearance
		4.2 Partial skeletonization, joints still together
	5	5.1 Gross skeletonization, some joints disarticulated
	6	6.1 Complete skeletonization
BDS	1	1.1 No visible changes
	2	2.1 Livor mortis, rigour mortis and vibices
	3	3.1 Grey to green discoloration
		3.2 Bloating with green discoloration and/or skin blisters, skin slippage and/or marbling
		3.3 Rectal purging of decompositional fluids
		3.4 Post-bloating: release of abdominal gasses with discoloration changing from green to black
	4	4.1 Decomposition of tissue producing sagging of flesh. Caving in of the abdominal cavity
		4.2 Skin having a leathery appearance
		4.3 Partial skeletonization, joints still together
	5	5.1 Gross skeletonization, some joints disarticulated
	6	6.1 Complete skeletonization
LDS	1	1.1 No visible changes
	2	2.1 Livor mortis, rigour mortis and vibices
		2.2 Discoloration: brownish shades particularly at the edges. Drying of fingers and toes
	3	3.1 Skin blisters and/or skin slippage and/or marbling
		3.2 Grey to green discoloration
		3.3 Brown to black discoloration
	4	4.1 Skin having a leathery appearance
		4.2 Partial skeletonization, joints and tendons still together
	5	5.1 Gross skeletonization, some joints disarticulated
	6	6.1 Complete skeletonization

The column ‘points’ represents the score given to the stadia. Point.1, point.2, etc. represent the different phenomena

*FDS* facial decomposition score, *BDS* body decomposition score, *LDS* limbs decomposition score

The first stage is called no visible signs. An individual just passed away and there are no decomposition signs visible yet. The second stage is where the livor mortis, rigour mortis, algor mortis and vibices occur. Drying of the body can lead to tache noir (when the eyes are open) and brownish discoloration starting at the edges (nose, ears, lips, fingers and toes) expands to the rest of the body [5]. Cloudy eyes can occur as soon as 10 min post-mortem if the eyes are open. If the eyes are shut, the cornea becomes cloudy after 24 h [37]. Tache noir can occur as soon as 1–2 h post-mortem [37]. The appearance of livor mortis is different for each human remain and can range between 15 min and many hours [37, 38]. It is also possible that it does not occur at all [38]. Rigour mortis is commonly detected between 3 and 6 hours. The face shows stiffening between 1 and 4 hours after death, and the limbs show stiffening between 3 and 6 hours after death [23, 28]. According to Galloway et al., livor mortis is followed by drying of the extremities [24]. Since the order in which the phenomena occur is variable, they were not given their own score.

The putrefaction, caused by autolysis, is not visible in stage two and starts in stage three with grey to green discoloration caused by the bacteria [39]. The same bacteria produce gasses which give the face and trunk a swollen appearance (bloating), starting in the areas with low turgor (eyelids, scrotum). The internal pressure caused by these gasses causes the tongue to protrude from the mouth, the abdomen to bloat and purging of putrefaction fluids out of the natural body openings (ears, nose, mouth and rectum) [25, 39]. These fluids leave a strong odour behind [25, 39]. The build-up pressure in the abdomen causes the blood to be pushed into the head, which causes the brown to black discoloration [40]. Also in this stage, skin blisters filled with decomposition fluids form and skin lets loose due to hydrolytic enzymes (slippage). Marbling is the result of gasses reacting with haemoglobin producing a greenish pigment in the veins [23, 39]. Further into this stage, the skin turns dark green to black [23, 38]. Phenomena as bloating, skin blisters and marbling can occur at the same time or one before the other in random order and rapidly after each other [10, 24]. Researchers disagree about the order in which the phenomena occur. Some researchers found bloating first and blistering and marbling later [12]. Some researchers found blistering and marbling first and later bloating [38, 40–42]. Some researchers found marbling first and later blisters and bloating [37, 43]. For this reason, these phenomena cannot be put in a rigid order.

Stage four starts when the putrefaction reaches an end and gasses are released and the tissue caves in, because tissues do not have the strength or the capability anymore, due to autolysis, to uphold connections. This gives the eyes, throat and abdominal cavity a hollow appearance [5]. Due to dehydration and draining of bodily fluids, the skin dries and gets a leathery appearance with discoloration and darkening of the skin. It becomes hard and contracts tightly around the bones [5]. The leathery skin forms a shell over the body and could protect the remaining decomposing tissue underneath [8, 24]. Also, partial skeletonization is visible, which means less than 50% bone is visible in that body area (face/neck, trunk or limbs). The joints are still together. According to Galloway et al., it is first caving in of the flesh followed by dehydration and the skin turning leathery. This in turn is followed by bone exposure (less than 50%) [24].

Stage five contains gross skeletonization which means most of the skin is gone and no soft tissue is left. More than 50% of bone is visible in that area which means either in the jaw, cheekbones, skull or a combination for the face/neck area. In the trunk, it could be the ribcage, the thoracic or lumbar vertebrae, the pelvic bones or a combination. In the limbs, it is a combination of the humerus, ulna, radius, wrist bones, the bones of the hand, the femur, the tibia or fibula or the bones of the feet. Some joints can be disarticulated such as the jaw, the cervical, thoracic or lumbar vertebrae, the ribcage or pelvic joints. In the limbs, it could be the shoulder, elbow, wrist, knee and ankle.

The last stage contains complete skeletonization (stage six) where only bones, cartilage, hair and sometimes a bit of skin are all that are left from the human remains [25, 39]. The skeletonization can be considered complete, when all soft tissue is removed [44].

**Fig. 2:**
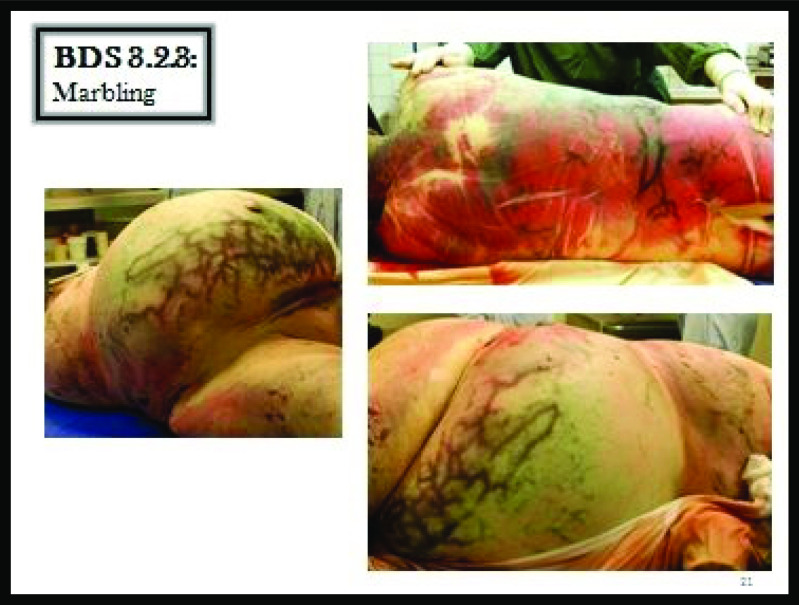
Body decomposition score 3.2.3: marbling

**Fig. 3:**
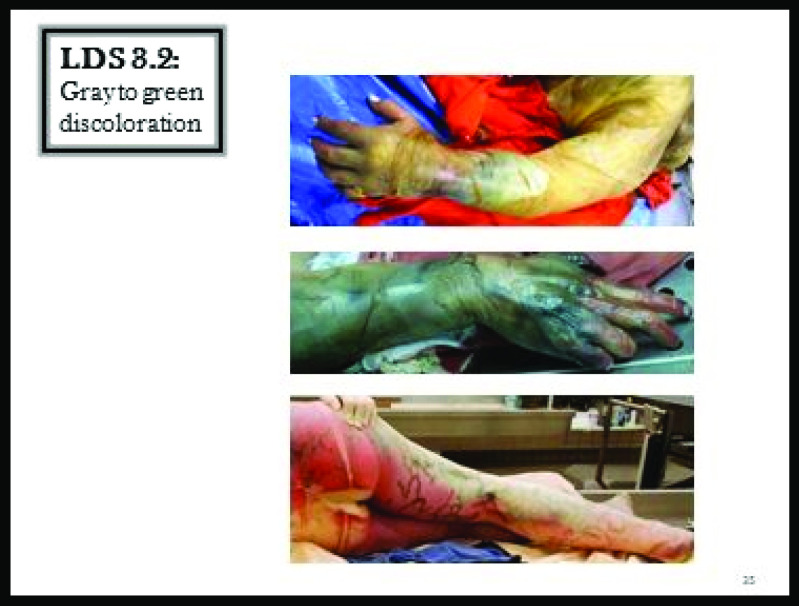
Limbs decomposition score 3.2: grey to green discoloration

The Book of Reference is a visual resource containing photographic examples of all the decomposition phenomena with their corresponding scores of the stage they are in. It contains a total of 84 specifically selected photographs. The photographs are placed in a chronological order, following the point-based system of the decomposition scoring method. Before each region is visually explained, the decomposition scoring method is shown in a table. Figures 2 and 3 are examples and part of the Book of Reference. Since stage 3.2 of both FDS and BDS and stage 3.1 of the LDS have multiple phenomena and all the phenomena have to be present in the Book of Reference, these stadia were further divided into subgroups, hence the reason for 3.2.3 in Fig. 2.

Twelve participants scored 45 test photographs. Table 2 shows the Fleiss kappa values. The lowest kappa score was 0.74 for the facial decomposition scored by the medical students (moderate agreement). The highest kappa score was 0.97 for the body decomposition score, also scored by the medical students (high agreement). In total, there are two values showing moderate agreement (FDS scored by students and LDS scored by forensic physicians) and seven values showing a high agreement [45].

**Table 2:** Results of the Fleiss kappa test

	Forensic physicians	Forensic scientists	Medical students
FDS	0.87	0.81	0.74
BDS	0.93	0.93	0.97
LDS	0.78	0.88	0.81

*FDS* facial decomposition score, *BDS* body decomposition score, *LDS* limbs decomposition score

### Estimating the post-mortem interval using the decomposition scoring method and ADD

Of the 91 cases with a known PMI, 28 individuals were female (30.8%) and 63 were male (69.2%) (see Appendix 1 for an overview of the characteristics, available online only). The age varied from 20 to 92 with a mean value of 54 (standard deviation (SD) ± 18). Most of the individuals died indoors (*n* = 79, 86.8%) and 12 individuals were found outside (13.2%). Twenty-nine cases were found in the Spring (31.9%), 19 in the Summer (20.9%), 21 in the Autumn (23.1%) and 22 in the Winter (24.2%).

The decomposition scores varied from score 3 to score 12. The median TDS is six, with an interquartile range (IQR) of six to nine. A TDS of six is by far the most common score (44%), which means a lot of individuals showed signs of livor mortis or rigour mortis. The second most common score is nine (18.7%). These individuals were in the putrefaction stage.

Of the 79 indoor cases, two individuals were found in a shed and one individual was found in a garage. Here, it was definitely unknown what the temperature was. It could not be decided if it was a normal inside temperature or a temperature close to the outside temperature. The ADD ranged from 4 to 1080 (*n* = 76). The median ADD is 28.5 with an IQR of 18 to 90. Of the 76 cases, 59 cases had an estimated mean temperature of 18 °C (77.6%). For the outdoor cases (*n* = 12), the ADD ranged from 3 to 512 with a median of 14.5 and an IQR of 8.3 to 19. In the 12 cases where the individuals were found outdoors, the weather station was as close as 0 km and as far as 29 with an IQR of 14 to 19 km [46].

**Fig. 4:**
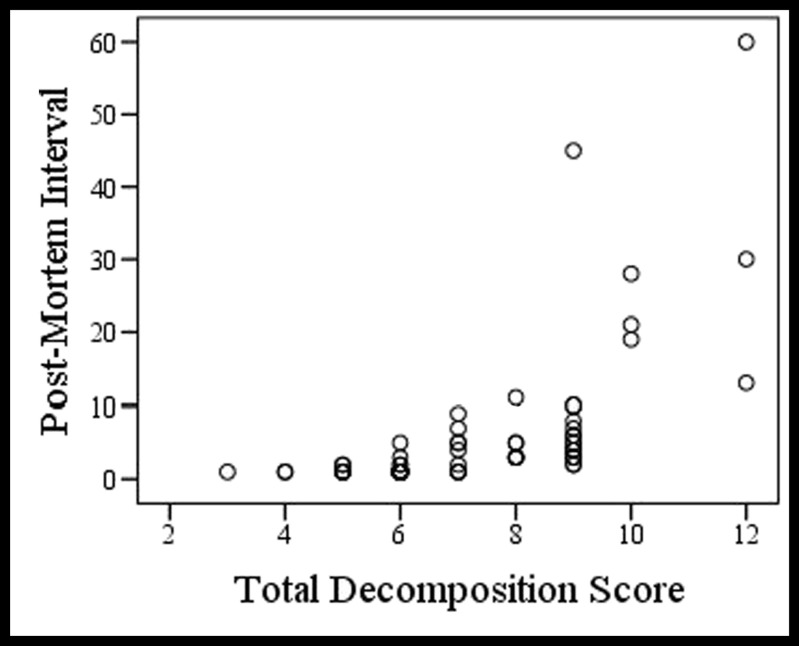
Scatter plot of the total decomposition score vs. the post-mortem interval in days (*n* = 91)

The correlation between the decomposition scores and PMI was the lowest between FDS and PMI (*r* = 0.697, *p* = 0.000, *n* = 91). The correlation between LDS and PMI was second lowest (*r* = 0.774, *p* = 0.000, *n* = 91) and the second highest correlation was between BDS and PMI (*r* = 0.801, *p* = 0.000, *n* = 91) (see Fig. 4). The highest correlation was found between TDS and PMI (*r* = 0.812, *p* = 0.000, *n* = 91). Even though the correlation between FDS and PMI is the lowest, it is still considered to be a strong correlation. The remaining three correlations are considered to be ‘very strong’ [47].

**Fig. 5:**
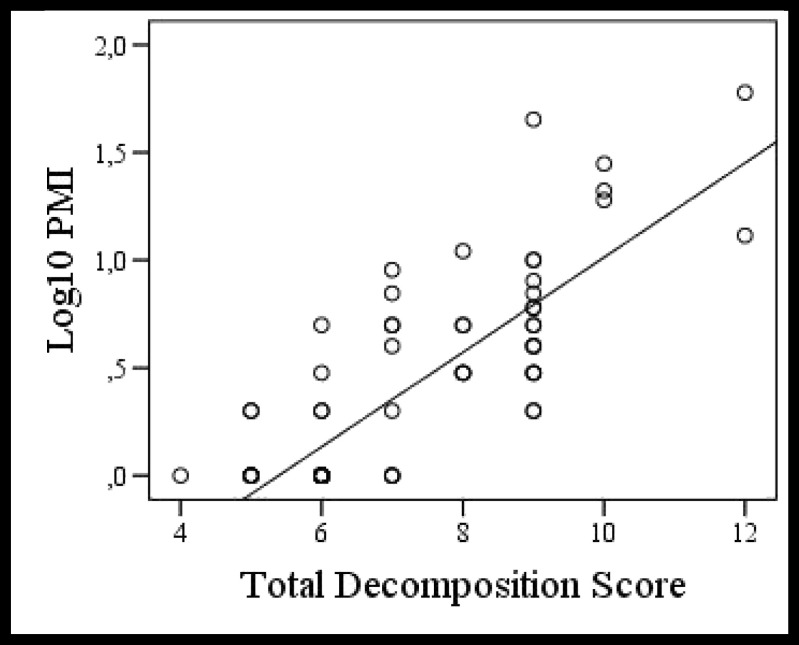
Plot of total decomposition score vs. log transformed post-mortem interval indoors. *R* ^2^ = 0.670 (*n* = 79)

**Fig. 6:**
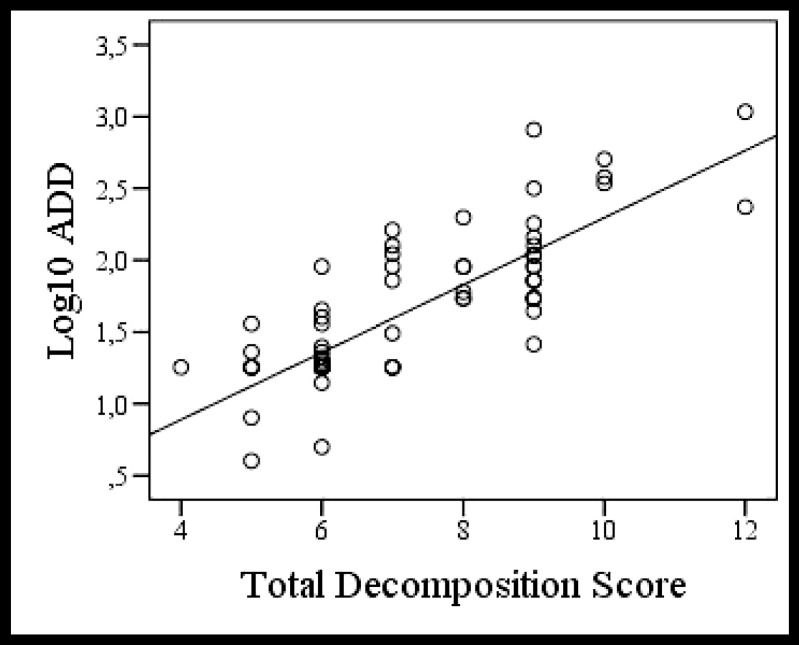
Plot of total decomposition score vs. log transformed accumulated degree days indoors. *R* ^2^ = 0.658 (*n* = 79)

**Fig. 7:**
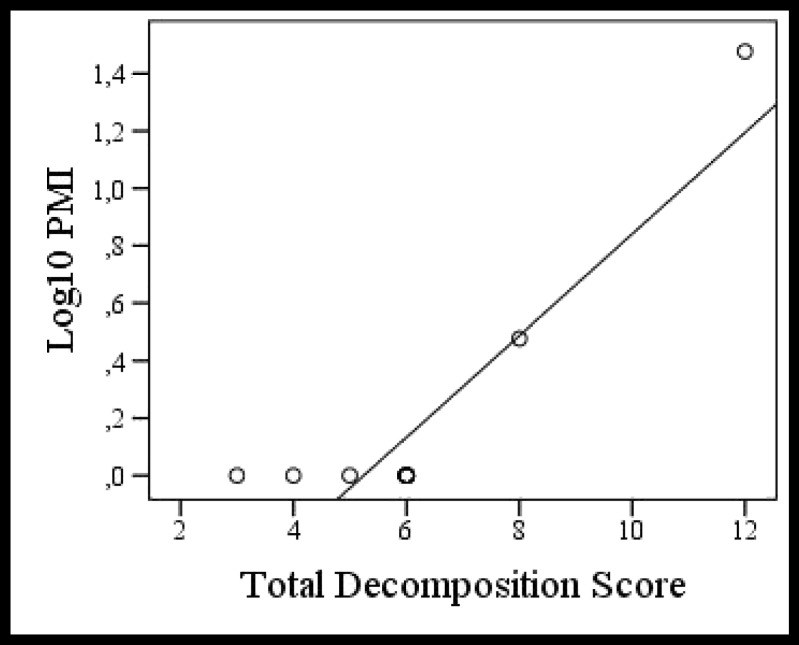
Plot of total decomposition score vs. log transformed post-mortem interval outdoors. *R* ^2^ = 0.803 (*n* = 12)

**Fig. 8:**
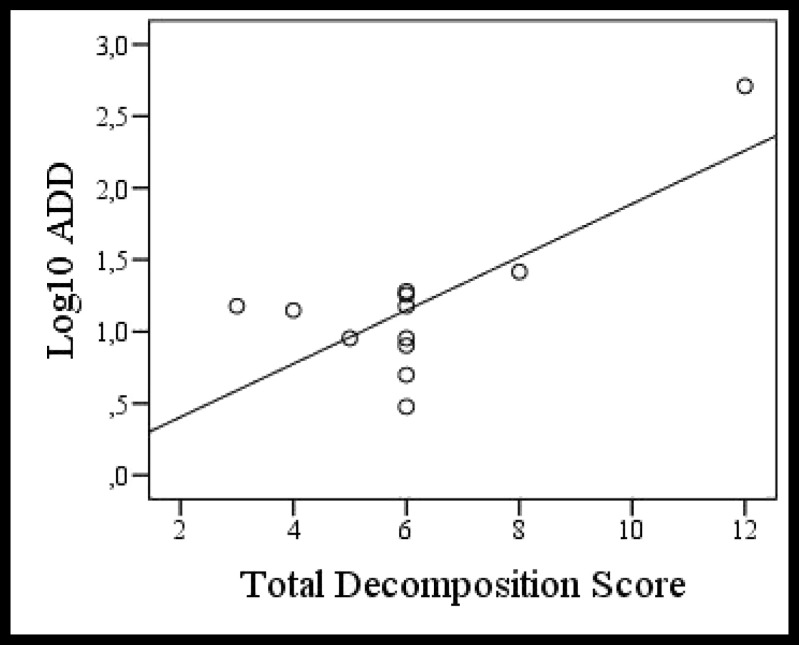
Plot of total decomposition score vs. log transformed accumulated degree days outdoors. *R* ^2^ = 0.557 (*n* = 12)

The relationship between PMI and TDS and ADD and TDS is exponential (curvilinear). The PMI and ADD were log transformed to achieve a linear relationship. After separating the indoor and outdoor cases, the following four formulas could be made with their associated standard errors. Starting with the indoor cases (*n* = 79), the TDS significantly estimated the PMI (*β* = 0.82, *p* = 0.000). The *R* ^2^ indicated that 67% of the variation in the PMI was estimated by the TDS. The developed formula is PMI = 10^(− 1.18 + 0.22·TDS) (see Fig. 5). The associated standard error of the regression in untransformed form (non-logged PMI) is 1.6 days. When using the TDS to estimate the ADD, the *R* ^2^ decreased to 66% (*β* = 0.81, *p* = 0.000). The developed formula is ADD = 10^(− 0.05 + 0.23·TDS) (see Fig. 6). The associated standard error of the regression in untransformed form (non-logged ADD) is 29.6 ADD. There were 12 cases outdoors. The TDS significantly estimated the PMI (*β* = 0.90, *p* = 0.000). The *R* ^2^ indicated that 80% of the variation in the PMI was estimated by the TDS. The developed formula is PMI = 10^(− 0.93 + 0.18·TDS) (see Fig. 7). The associated standard error of the regression in untransformed form (non-logged PMI) is 2.9 days. When using the TDS to estimate the ADD, the *R* ^2^ decreased to 56% (*β* = 0.75, *p* = 0.005). The developed formula is ADD = 10^(0.03 + 0.19·TDS) (see Fig. 8). The associated standard error of the regression in untransformed form (non-logged ADD) is 52.1 ADD. The standard errors were calculated with the *t* distribution (see Table 3).

**Table 3:** Developed formula for indoor and outdoor cases

	*R* ^2^	*β*	*p*	Formula	SE
Indoors, PMI	0.670	0.82	0.000	PMI = 10^(−1.18 + 0.22·TDS)	1.6 days
Indoors, ADD	0.658	0.81	0.000	ADD = 10^(−0.05 + 0.23·TDS)	29.6 ADD
Outdoors, PMI	0.803	0.90	0.000	PMI = 10^(−0.93 + 0.18·TDS)	2.9 days
Outdoors, ADD	0.557	0.75	0.005	ADD = 10^(0.03 + 0.19·TDS)	52 ADD

*PMI* post-mortem interval, *ADD* accumulated degree days, *TDS* total decomposition score, *R* ^2^ determination coefficient, *β* standardised coefficient, *p p* value, statistically significant as *p* < 0.05, *SE* standard error

## Discussion

### Decomposition scoring method

The developed scoring method in this study has high interrater reliability scores and proved to be a valid method to score the decomposition of human remains found on land. It is a simple, practical and user friendly method for fast decomposition scoring in the field. An autopsy is not required, which is a huge advantage since this is a right solely for the (forensic) pathologists in the Netherlands. The method can be used to score the decomposition of human remains found on land in all geographic regions with a similar climate as in the Netherlands (a temperate climate with a warm summer without a dry season (Köppen-Geiger classification ‘Cfb’)) [48–50].

The developed decomposition scoring method in this research uses the new terminology derived from the terms introduced by De Kat et al., who developed an aquatic decomposition scoring method [32]. The human remain is divided into three body parts called facial aquatic decomposition score (FADS), body aquatic decomposition score (BADS) and limbs aquatic decomposition score (LADS). The phenomena in our developed decomposition scoring method were not given their own scores, but rather put together in a stage. Phenomena can occur at the same time or one by one in random order and rapidly after each other [10, 12, 24, 37, 40]. They cannot be put in a rigid order, because this order does not exist in the decomposition process. As a result, the maximum TDS was 18 instead of 32.

Multiple researchers studied the interrater reliability of the decomposition scoring method made by Megyesi et al. [5]. Nawrocka et al. [28] and Dabbs et al. [27] both found high values of interrater reliability when using the original total body score (TBS) (respectively, Krippendroff’s *α* = 0.818 and absolute correlation coefficient of 0.990). Both researches made suggestions for improvement of the original decomposition scoring method so that even higher results could be achieved. We relate to some of these, for instance, the percentages used for skeletonization in stage four and five (respectively, less than 50% bone visible and more than 50% bone visible). Nawrocka et al. suggested that the percentages should be replaced by the determination of particular bones which are exposed [28]. In our developed decomposition scoring method, the percentages stayed, but the determination of bones was added in the explanation of the scoring method. Dabbs et al. [27] noted colours during the decomposition of human remains which are not accounted for in the original decomposition scoring method of Megyesi et al. [5]. We changed the termination ‘pink-white appearance’ to ‘livor mortis’, but the fact remains that it is difficult to objectivise in people with darker skin tones. There were also bodies with orange to brown, leathery skin in our study, but the colour was not specified in our scoring system. Dabbs et al. [27] also found bloating in the limbs. In our literature search, this was not found, hence the reason this phenomenon was not added in the scoring method.

Participants in our study announced minor restrictions of the method developed in this research. Not everything from a body part was visible on the photographs. As a result, the decomposition could be further along than is visible on the photographs. Scoring the human remains could be more reliable when the body can be fully investigated. Second was seeing the difference between dried and leathery fingers and toes. It was said that the skin needed to be touched to make the optimal decision and to make the difference between score two and score four.

### Estimating the post-mortem interval using the decomposition scoring method and ADD

The second aim of this research was to find a practical way to estimate the post-mortem interval. The developed method consists of the developed decomposition scoring method, a Book of Reference and a formula. Before the formulas were developed, the indoor and outdoor cases were separated, because outdoor bodies are influenced by more factors than indoor bodies, which alters the decomposition rate [14]. It is believed that human remains found indoors are more ‘protected’ from external factors and temperature is not as variable as outside. The results show that the variation in PMI in different cases can be estimated by the TDS with respectively 67% (indoor cases) and 80% (outdoor cases). Using the TDS to estimate the ADD, the estimation decreased to respectively 66 and 56%. Reasons for this decrease could be the following: The temperature for the cases found indoors was largely estimated. Of the 76 cases, 59 were estimated to have a mean daily temperature of 18 °C. It is most likely that this estimation is not accurate in all the included cases and this creates a margin of error. It is difficult to obtain the exact temperature during the day and during the time a body is decomposing [51]. To obtain the actual daily temperature(s), it has to be known when an individual died so the data recording can be started. This is also the greatest problem because when a body is found, the greatest question is when the individual died. In the outdoor cases, there are other possible temperature errors. The ADD is the sum of mean temperatures. At 1 day, there can be a difference of 10° in minimum and maximum temperature in the Netherlands [35] which in turn influences the decomposition process. The weather stations were as close as 0 km and as far as 29 km from the discovered bodies. Possible micro-climatic differences could influence the decomposition rate. Thereby, the distinction between woods and open field was not made in this research and this could mean differences in sun exposure (shadow or full exposure) and thus (carcass) temperature. The carcass temperature can also be higher than expected because of the heat generated by insects while decomposing human remains and which the ADD does not account for [9, 10, 52]. Due to this heat, the internal carcass temperature can be higher than the ambient temperature [25, 39].

In this research, temperatures below zero were not recorded as negative values, but as zero. It is unknown at which temperature putrefaction stops. Micozzi [36] states that no putrefaction occurs at temperatures below 4 °C while Vass et al. [22] states it is below 0 °C. Since it is unknown, the wrong value of ADD could be used in studies.

A possible bias in this research is the post-mortem interval. This interval is based on bystanders who were present at the time of death (for example ambulance personnel), last used newspaper or tv-guide, the mail in the mailbox, entomology, the last time a person was in contact or was seen or the last time an individual used social media. In the Netherlands, the PMI is found based on multiple factors combined, but the estimated PMI will always have a margin of error.

Megyesi et al. [5] included cases in which the PMI was based on entomology findings, because it is said that this is one of the most accurate ways to determine the PMI [19, 25]. These cases (*n* = 48) with an estimated PMI were included in the original regression analysis. Moffat et al. [53] excluded these cases in their method to improve the original equation of Megyesi et al. [5] because there are potential errors involved using entomology to estimate the post-mortem interval [54, 55]. The 95% confidence interval was calculated using the standard error (SE). The SE found with the *t* distribution is much smaller in this research (see Table 3), compared to the SE used by Megyesi et al. (388.16 ADD). The 95% prediction interval would have been 787.71 ADD, but instead Megyesi doubled the SE first to 787.71 ADD and then used it as a 95% prediction interval, which became ± 787.71 ADD. This means the interval is larger than it should be and much larger than the 95% prediction interval used in this study. Suckling et al. [8] used the equation provided by Megyesi et al. [5] and the results showed that the used 95% prediction interval (± 787.71 ADD) was not small enough to provide a precise estimation of the PMI.

The formulas developed in this research estimate the variation in PMI between 56 and 80% (see Table 3). We investigated in which cases the estimated PMI was accurate and in which cases it was not. In 23 cases, the PMI calculated with the formulas did not fall in the 95% confidence interval. Of these 23 cases, 13 cases had a PMI under 10 days and the TDS ranged between 6 and 9. In the other ten cases, the PMI ranged between 10 and 60 days and the TDS between 8 and 12. It seems that a combination of a high TDS and a short PMI (for example PMI of 2 days and a TDS of 9) does not give an accurate estimation. In all the cases with an actual PMI of 10 days or longer (*n* = 10), the formulas do not give an accurate approximation of the PMI. Caution is advised when drawing conclusions based on the formulas developed in this research, especially when the PMI is long.

## Conclusions

In this research, it can be concluded that the developed decomposition scoring method is a practical and user friendly method to measure decomposition for human remains found on land with a high interrater reliability. It can be used in all geographic regions with a similar climate as in the Netherlands. Second, the TDS has a strong correlation with time and the PMI can be estimated using the developed decomposition scoring method, but caution is advised in cases where the PMI is long. As for temperature, the ADD does not account for all the heat present in decomposing remains and this is a possible bias.

## Electronic supplementary material

ESM 1 (DOCX 23 kb)

## References

[1] Cockle DL, Bell LS (2015). Human decomposition and the reliability of a ‘Universal’ model for post mortem interval estimations. Forensic Sci Int.

[2] Henßge C, Madea B (2004). Estimation of the time since death in the early post-mortem period. Forensic Sci Int.

[3] Warther S, Sehner S, Raupach T, Püschel K, Anders S (2012). Estimation of the time since death: post-mortem contractions of human skeletal muscles following mechanical stimulation (idiomuscular contraction). Int J Legal Med.

[4] Madea B, Madea B (2016). Supravitality in tissues. Estimation of the time since death.

[5] Megyesi MS, Nawrocki SP, Haskell NH (2005). Using accumulated degree-days to estimate the postmortem interval from decomposed human remains. J Forensic Sci.

[6] Sutherland A, Myburgh J, Steyn M, Becker PJ (2013). The effect of body size on the rate of decomposition in a temperate region of South Africa. Forensic Sci Int.

[7] Matuszewski S, Konwerski S, Fratczak K, Szafalowicz M (2014). Effect of body mass and clothing on decomposition of pig carcasses. Int J Legal Med.

[8] Suckling JK, Spradley MK, Godde K (2015). A longitudinal study on human outdoor decomposition in Central Texas. J Forensic Sci.

[9] Johnson AP, Mikac KM, Wallman JF (2013). Thermogenesis in decomposing carcasses. Forensic Sci Int.

[10] Campobasso CP, Di Vella G, Introna F (2001). Factors affecting decomposition and Diptera colonization. Forensic Sci Int.

[11] Mann RW, Bass WM, Meadows L (1990). Time since death and decomposition of the human body: variable and observations in case and experimental field studies. J Forensic Sci.

[12] Damann FE, Carter DO, Pokines JT, Symes SA (2014). Human decomposition ecology and postmortem microbiology. Manual of forensic taphonomy.

[13] Bass WM, Haglund WD, Sorg MH (1996). Outdoor decomposition rates in Tennessee. Forensic taphonomy: the postmortem fate of human remains.

[14] Simmons T, Adlam RE, Moffatt C (2010). Debugging decomposition data—comparative taphonomic studies and the influence of insects and carcass size on decomposition rate. J Forensic Sci.

[15] Payne JA (1965) A summer carrion study of the baby pig *Sus scrofa* Linnaeus. Ecology 45(5):592–602. 10.2307/1934999

[16] Anderson GS (2000). Minimum and maximum development rates of some forensically important *Calliphoridae* (*Diptera*). J Forensic Sci.

[17] Dautartas AM (2009) The effect of various coverings on the rate of human decomposition. Master’s Thesis, University of Tennessee

[18] Zhou C, Byard RW (2011). Factors and processes causing accelerated decomposition in human cadavers—an overview. J Forensic Leg Me.

[19] Spicka A, Johnson R, Bushing J, Higley LG, Carter DO (2011). Carcass mass can influence rate of decomposition and release of ninhydrin-reactive nitrogen into gravesoil. Forensic Sci Int.

[20] Kelly JA, van der Linde TC, Anderson GS (2009). The influence of clothing and wrapping on carcass decomposition and arthropod succession during the warmer seasons in Central South Africa. J Forensic Sci.

[21] Voss SC, Cook DF, Dadour IR (2011). Decomposition and insect succession of clothed and unclothed carcasses in Western Australia. Forensic Sci Int.

[22] Vass AA, Bass WM, Wolt JD, Foss JE, Ammons JT (1992). Time since death determinations of human cadavers using soil solution. J Forensic Sci.

[23] Payne-James J, Jones R, Karch SB, Manlove J (2011). Simpson’s forensic medicine.

[24] Galloway A, Birkby WH, Jones AM, Henry TE, Parks BO (1989). Decay rates of human remains in an arid environment. J Forensic Sci.

[25] Anderson GS, VanLaerhoven SL (1996). Initial studies on insect succession on carrion in southwestern British Columbia. J Forensic Sci.

[26] Shalaby OA, deCarvalho LML, Goff ML (2000). Comparison of patterns of decomposition in a hanging carcass and a carcass in contact with soil in a xerophytic habitat on the Island of Oahu, Hawaii. J Forensic Sci.

[27] Dabbs GR, Connor M, Bytheway JA (2016) Interobserver reliability of the total body score system for quantifying human decomposition. J Forensic Sci 61(2):445–451. 10.1111/1556-4029.12962 10.1111/1556-4029.12962 27404618

[28] Nawrocka M, Fratczak K, Matuszewski S (2016). Inter-rater reliability of total body score—a scale for quantification of corpse decomposition. J Forensic Sci.

[29] Randolph JJ. Online Kappa Calculator. http://justus.randolph.name/kappa; 2008 Access 16.01.26

[30] Rice PL, Orgill DP. Classification of burns. https://www.uptodate.com.proxy-ub.rug.nl/contents/classification-of-burns?source=search_result&search=rule+of+nine&selectedTitle=1~5#H16; 2016 Access 16.03.21

[31] Bijl D, Semmekrot B, van Loenen A, Bindels PJE, Kneepkens CMF (2013). Farmacotherapie. Kindergeneeskunde.

[32] van Daalen MA, de Kat DS, Oude Grotebevelsborg BFL, Warnaar J, Oostra RJ, Duijst-Heesters WLJM (2017) An aquatic decomposition scoring method to potentially predict the postmortem submersion interval of bodies recovered from the North Sea. J Forensic Sci 62(2):369–373. 10.1111/1556-4029.13258 10.1111/1556-4029.13258 28247448

[33] Rodriguez WC, Haglund WD, Sorg MH (1996). Decomposition of buried and submerged bodies. Forensic taphonomy: the postmortem fate of human remains.

[34] Vass AA (2011). The elusive universal post-mortem interval formula. Forensic Sci Int.

[35] Koninklijk Nederlands Meteorologisch Instituut, Klimatologie—Daggegevens van het weer in Nederland. http://projects.knmi.nl/klimatologie/daggegevens/selectie.cgi; Accessed 15.12.12 until 16.02.22

[36] Micozzi MS, Haglund WD, Sorg MH (1996). Frozen environments and soft tissue preservation. Forensic taphonomy: the postmortem fate of human remains.

[37] Madea B, Henssge C, Reibe S, Tsokos M, Kernbach-Wighton G, Madea B (2014). Postmortem changes and time since death. Handbook of forensic medicine.

[38] Knight B, Saukko P (2004). Knight’s forensic pathology.

[39] Lee Goff M (2009). Early post-mortem changes and stages of decomposition in exposed cadavers. Exp Appl Acarol.

[40] Reijnders UJL, Das C (2007). De lijkschouw in de praktijk.

[41] Micozzi MS (1991). Postmortem change in human and animal remains.

[42] Bucholtz A (2015). Death investigation.

[43] Van Voorde W (2016) Forensische Geneeskunde, 3^rd^ ed. die Keure, Brugge

[44] Clark MA, Worrell MB, Pless JE, Haglund WD, Sorg MH (1996). Postmortem changes in soft tissues. Forensic taphonomy: the postmortem fate of human remains.

[45] Marais-Werner A (2016) Decomposition patterns of buried remains in the central Highveld region of South Africa. Dissertation, University of Pretoria 10.1177/0025802417705263 28480785

[46] Afstand berekenen. https://nl.afstand.org/. Accessed 17.01.30

[47] Statstutor. Spearman’s correlation. https://www.statstutor.ac.uk/resources/uploaded/spearmans.pdf. Accessed 16.02.24

[48] Weatherbase. Flagstaff, Arizona. https://www.weatherbase.com/weather/weather-summary.php3?s=557327&cityname=Flagstaff%2C+Arizona%2C+United+States+of+America&units. Accessed 17.01.29

[49] Visit Arizona. Arizona Weather. https://www.visitarizona.com/arizona-travel-info/arizona-weather. Accessed 17.01.29

[50] Peel MC, Finlayson BL, McMahon TA (2007). Updated world map of the Köppen-Geiger climate classification. Hydrol Earth Syst Sci Discuss.

[51] Hayman J, Oxenham M (2016). Human body decomposition.

[52] Bonacci T, Brandmayr P, Greco S, Tersaruolo C, Vercillo V, Brandmayr TZ (2010). A preliminary investigation of insect succession on carrion in Calabria (southern Italy). Terr Arthropod Rev.

[53] Moffatt C, Simmons T, Lynch-Aird J (2016). An improved equation for TBS and ADD: establishing a reliable postmortem interval framework for casework and experimental studies. J Forensic Sci.

[54] Villet MH, Richards CS, Midgley JM, Amendt J, Campobasso CP, Goff ML, Grassberger M (2010). Contemporary precision, bias and accuracy of minimum post-mortem intervals estimated using development of carrion-feeding insects. Current concepts in forensic entomology.

[55] Anderson GS, Byrd JH, Castner JL (2001). Insect succession on carrion and its relationship to determining time of death. Forensic entomology: the utility of arthropods in legal investigations.

