# Supplementary material for: The development of a post-mortem interval estimation for human remains found on land in the Netherlands
Source: Int J Legal Med. 2017 Nov 6;132(3):863–73. doi: 10.1007/s00414-017-1700-9 (PMC5920129; doi:10.1007/s00414-017-1700-9)
Supplement: Supplementary file 1 [file 414_2017_1700_MOESM1_ESM.docx]

Overview of the characteristics of 91 cases

| **Case number** | **Gender**  **M/F** | **Indoors Outdoors** | **PMI^∆^** | **PMIꜞ** | **ADD** | **TDS** | **PMI calculated** | **ADD**  **calculated** |
| --- | --- | --- | --- | --- | --- | --- | --- | --- |
| 1 | F | I | 2 | 9 | 36 | 5 | 0.8 | 12.6 |
| 2 | M | I | 1 | 9 |  | 5 | 0.8 | 12.6 |
| 3 | M | I | 1 | 9 | 18 | 6 | 1.4 | 21.4 |
| 4 | M | O | 1 | 9 | 14 | 4 | 0.6 | 6.2 |
| 5 | F | I | 1 | 9 | 18 | 6 | 1.4 | 21.4 |
| 6 | M | I | 11 | 7 | 198 | 8 | 3.8 | 62.7 |
| 7 | M | I | 1 | 0 | 18 | 5 | 0.83 | 12.6 |
| 8 | M | I | 5 | 9 | 90 | 9 | 6.3 | 104.7 |
| 9 | M | O | 30 | 9 | 512 | 12 | 17 | 204.2 |
| 10 | M | I | 45 | 4/5 | 810 | 9 | 6.3 | 104.7 |
| 11 | F | I | 1 | 0 | 18 | 5 | 0.83 | 12.6 |
| 12 | M | I | 7 | 9 | 126 | 9 | 6.3 | 104.7 |
| 13 | F | I | 5 | 9 | 90 | 6 | 1.4 | 21.4 |
| 14 | M | I | 1 | 0 | 18 | 6 | 1.4 | 21.4 |
| 15 | F | I | 1 | 0 | 18 | 6 | 1.4 | 21.4 |
| 16 | M | I | 1 | 0 | 4 | 5 | 0.83 | 12.6 |
| 17 | M | I | 10 | 9 | 315 | 9 | 6.3 | 104.7 |
| 18 | F | I | 13 | 6 | 234 | 12 | 28.8 | 512.9 |
| 19 | F | I | 5 | 9 | 110 | 7 | 2.3 | 36.3 |
| 20 | M | I | 9 | 4 | 162 | 7 | 2.3 | 36.3 |
| 21 | M | I | 3 | 5 | 60 | 8 | 3.8 | 61.7 |
| 22 | M | I | 6 | 0 | 108 | 9 | 6.3 | 104.7 |
| 23 | M | I | 5 | 0 | 90 | 8 | 3.8 | 61.7 |
| 24 | M | I | 4 | 8 | 72 | 9 | 6.3 | 104.7 |
| 25 | M | I | 3 | 9 | 54 | 8 | 3.8 | 61.7 |
| 26 | F | I | 2 | 0 | 44 | 9 | 6.3 | 104.7 |
| 27 | M | I | 60 | 9 | 1080 | 12 | 28.8 | 512.9 |
| 28 | F | I | 7 | 0/4 | 126 | 7 | 2.3 | 36.3 |
| 29 | M | O | 3 | 9 | 26 | 8 | 3.2 | 35.5 |
| 30 | F | I | 1 | 9 | 18 | 7 | 2.3 | 36.3 |
| 31 | M | I | 5 | 4 | 90 | 8 | 3.8 | 61.7 |
| 32 | M | I | 3 | 9 | 54 | 9 | 6.3 | 104.7 |
| 33 | F | I | 4 | 0 | 72 | 9 | 6.3 | 104.7 |
| 34 | M | I | 1 | 9 | 18 | 5 | 0.8 | 12.6 |
| 35 | M | O | 1 | 9 | 8 | 6 | 1.4 | 14.8 |
| 36 | M | I | 3 | 0 | 54 | 9 | 6.3 | 104.7 |
| 37 | F | I | 5 | 0 | 90 | 7 | 2.3 | 36.3 |
| 38 | M | I | 4 | 9 | 72 | 7 | 2.3 | 36.3 |
| 39 | F | I | 1 | 0 |  | 6 | 1.4 | 21.4 |
| 40 | M | I | 8 | 9 | 144 | 9 | 6.3 | 104.7 |
| 41 | M | I | 1 | 9 | 18 | 6 | 1.4 | 21.4 |
| 42 | M | I | 4 | 9 | 72 | 9 | 6.3 | 104.7 |
| 43 | M | O | 1 | 0 | 15 | 6 | 1.4 | 14.8 |
| 44 | M | I | 1 | 9 | 18 | 6 | 1.4 | 21.4 |
| 45 | M | O | 1 | 2 | 9 | 5 | 0.9 | 14.8 |
| 46 | F | I | 2 | 0 | 36 | 6 | 1.4 | 21.4 |
| 47 | M | I | 1 | 3 | 18 | 4 | 0.5 | 7.4 |
| 48 | M | I | 2 | 0 | 31 | 7 | 2.3 | 36.3 |
| 49 | M | I | 1 | 0 | 18 | 6 | 1.4 | 21.4 |
| 50 | M | I | 2 | 0 | 4 | 5 | 0.83 | 12.6 |
| 51 | M | I | 1 | 0 | 18 | 6 | 1.4 | 21.4 |
| 52 | M | I | 1 | 0 | 21 | 6 | 1.4 | 21.4 |
| 53 | F | I | 1 | 0 | 23 | 6 | 1.4 | 21.4 |
| 54 | M | I | 1 | 0 | 18 | 6 | 1.4 | 21.4 |
| 55 | M | I | 3 | 5 | 54 | 9 | 6.3 | 104.7 |
| 56 | M | I | 6 | 0/4 | 108 | 9 | 6.3 | 104.7 |
| 57 | M | O | 1 | 0 | 5 | 6 | 1.4 | 14.8 |
| 58 | M | I | 1 | 9 | 18 | 6 | 1.4 | 21.4 |
| 59 | M | I | 1 | 9 | 18 | 6 | 1.4 | 21.4 |
| 60 | M | I | 1 | 9 | 18 | 6 | 1.4 | 21.4 |
| 61 | F | I | 1 | 9 | 23 | 5 | 0.83 | 12.6 |
| 62 | M | I | 3 | 9 | 45 | 6 | 1.4 | 21.4 |
| 63 | M | I | 3 | 9 | 54 | 8 | 3.8 | 61.7 |
| 64 | F | I | 10 | 9 | 180 | 9 | 6.3 | 104.7 |
| 65 | M | I | 21 | 9 | 378 | 10 | 10.5 | 177.8 |
| 66 | F | I | 28 | 9 | 504 | 10 | 10.5 | 177.8 |
| 67 | F | I | 2 | 0 | 40 | 6 | 1.4 | 21.4 |
| 68 | M | I | 1 | 9 | 25 | 6 | 1.4 | 21.4 |
| 69 | M | I | 1 | 0 | 14 | 6 | 1.4 | 21.4 |
| 70 | M | I | 1 | 9 | 5 | 6 | 1.4 | 21.4 |
| 71 | M | I | 1 | 9 | 18 | 6 | 1.4 | 21.4 |
| 72 | M | I | 1 | 9 | 18 | 7 | 2.3 | 36.3 |
| 73 | F | I | 1 | 0 | 18 | 6 | 1.4 | 21.4 |
| 74 | F | I | 19 | 4 | 342 | 10 | 10.5 | 177.8 |
| 75 | F | I | 1 | 2 | 18 | 6 | 1.4 | 21.4 |
| 76 | M | I | 1 | 0 | 19 | 6 | 1.4 | 21.4 |
| 77 | F | O | 1 | 9 | 9 | 6 | 1.4 | 14.8 |
| 78 | M | O | 1 | 2 | 18 | 6 | 1.4 | 14.8 |
| 79 | M | I | 2 | 1 | 26 | 9 | 6.3 | 104.7 |
| 80 | F | I | 1 | 0 | 18 | 6 | 1.4 | 21.4 |
| 81 | F | I | 1 | 1 |  | 6 | 1.4 | 21.4 |
| 82 | M | I | 1 | 2 | 18 | 6 | 1.4 | 21.4 |
| 83 | M | I | 1 | 1 | 18 | 6 | 1.4 | 21.4 |
| 84 | M | O | 1 | 2 | 15 | 3 | 0.4 | 4 |
| 85 | M | I | 1 | 9 | 18 | 6 | 1.4 | 21.4 |
| 86 | F | I | 1 | 0 | 18 | 6 | 1.4 | 21.4 |
| 87 | F | I | 1 | 0 | 20 | 6 | 1.4 | 21.4 |
| 88 | M | O | 1 | 2 | 19 | 6 | 1.4 | 14.8 |
| 89 | F | I | 1 | 9 | 18 | 7 | 2.3 | 36.3 |
| 90 | M | O | 1 | 2 | 3 | 6 | 1.4 | 14.8 |
| 91 | M | I | 5 | 0 | 90 | 9 | 6.3 | 104.7 |

PMI^∆^ = the actual Post-Mortem Interval in days reported in the case file, PMIꜞ = Post-Mortem Interval estimation based on: 0 = Last known contact, 1 = Last seen, 2 = Emergency services, 3 = Detention Caretakers, 4 = TV-guide, 5 = Newspapers, 6 = Mail, 7 = Entomology, 8 = Medication, 9 = Unknown.

ADD = Accumulated Degree Days based on the actual PMI^∆^.

PMI calculated = the Post-Mortem Interval calculated with the developed formulas.

ADD calculated = Accumulated Degree Days calculated with the developed formulas.
